# Treatment of Diseases of the Hair

**Published:** 1877-09

**Authors:** 


### Selections.

### TREATMENT OF DISEASES OF THE HAIR.

(From The Doctor, August, 1877.)

Professor Erasmus Wilson has devoted this year's course on Dermatology to the diseases of the hair system. Among these, baldness and greyness occupies an important position, and like others, were discussed in an exhaustive manner. The lectures that are appearing *in extenso* in the *Medical Examiner* will be perused by all dermatologists, and therefore we may safely confine ourselves to the therapeutics of baldness, alopecia, or calvities, and baldness. Poliothrix, or canities, as a prophylactic brushing is of the first importance, as it promotes circulation and renovation, as well as removes scurf. Though advocating cleanliness to the utmost, to the question, "Am I to wash my head?" Mr. Wilson replies emphatically "No."

Water is a less effective stimulant than brushing, even when sea-water is used. Cutting is less effective in promoting growth than is commonly believed, but is advantageously applied to the short, slender hairs commonly called "young hairs," and which are so often associated with baldness.

The number of applications warranted to cure baldness is legion, and their constant failure familiar to all. From the fat of the bear to the spirituous essence, most of them are stimulants, as is the petroleum lately vaunted. All occasionally seem to succeed—that is, the public observe that the hair grows after the use, and the public never experiences the fallacy of *post hoc ergo propter hoc* in matters therapeutic. Our readers will be glad to hear the experience of Professor Wilson on the use of some of these remedies, which we proceed to give, beginning with cantharides, acetic acid, and ammonia. He found croton oil, although excellent as to its stimulating effects, peculiarly unmanageable as an application for the head. In one instance, in a young lady, it produced so much redness and swelling, symptoms which extended to the eyelids and face, that he was threatened with a legal prosecution for prescribing it. In a few days the swelling subsided, and as the hair grew again satisfactorily, he was let off without action or thanks. The croton oil having played a similar prank on a second occasion, he has discarded it.

The milder and more manageable stimulation of cantharides has gained it a popular character, both as a stimulant and as an epispastic; but it also is not without its reverse. It is too apt to stretch its stimulative power to the extent of irritation, to give rise to inflammatory congestion and vesication, and sometimes to suppuration and ulceration. Every now and then it may come across a peculiarly sensitive skin, while at other times it may have been employed too energetically, both as to quantity and time. Mr. Wilson has seen several instances in which cantharadine has been absorbed into the system, and has given rise to ischuria. As a rule, therefore, he rarely uses cantharides, and then always in a guarded manner. Certainly it is not a remedy to be trusted to the acknowled indiscretion of the public as a popular remedy.

Acetic acid, or rather strong pyroligneous acid, he has discontinued for many years. But judging from the reports of those who have employed it, it is still a favorite, although its favor is somewhat alloyed by the convicting evidence of a strong and disagreeable odor.

Ammonia is Mr. Wilson's favorite; it may be limited easily to the bounds of stimulation; it is unlikely to create inflammation and its consequences; it is neither absorbable into the system, nor could it do harm if such were the case, and its odor, refreshing at the moment of its use, speedily evaporates. A formula for an ammonia lotion is to be found in Willan and Bateman's book; ammonia is the active principle of a lotion for the hair of considerable reputation, called Locock's lotion; and it is also the essential ingredient of a lotion which is sold through the length and breadth of the kingdom under his name, and probably taken from some of his works.

Now, in a case of ordinary madesia or defluvium capillorum, the popular falling out of the hair, he still prescribes a lotion composed of strong liquor ammoniæ, almond oil, and chloroform, of each one part, diluted with five parts of spirits of wine, or spirits of rosemary, and made pleasant as to fragrancy by the addition of a drachm of the essential oil of lemons. The instructions for the use of this lotion are, that it should be dabbed upon the skin of the head after thorough friction with the hair-brush. No doubt there are cases in which this lotion must be used with caution. It may be diluted if necessary; it may be applied sparingly or abundantly; and it may be used daily or otherwise.

There are cases in which a less stimulating and even a refrigerating lotion may be required, and where an objection may be raised to the quantity of oil contained in the former lotion, in which cases a lotion of borax and glycerine, two drachms of each to eight ounces of distilled water, is cooling and refreshing; this lotion allays dryness of the skin, removes scurf, and subdues irritability.

In cases of complete baldness, the phalachrosis of the Greeks, and also in alopecia areata, a stronger stimulant application will be required. In such a case he recommends frictions with a liniment composed of equal parts of the liniments of camphor, ammonia, chloroform, and aconite, to be well rubbed into the bare places daily, or even twice a day, so as to produce a moderate amount of stimulation. In cases of ophiasis, due to neuralgia of the cutaneous nerves of the scalp, this liniment is very valuable. In other cases the liniment of iodine may be painted on the bare patches daily, or they may be stimulated by friction with the ointment of cantharides, or any other powerful stimulant. Painting the discs of area with the epispastic fluid of the Pharmacopœia may also occasionally be resorted to, or the epispastic fluid may be diluted with spirits of camphor. The intention of all these local remedies is to stimulate without setting up irritation; to increase the energy of circulation and innervation of the part, and in some instances to abstract the excess of fluids from the tissues of the skin by inducing exudation. But these results must be accomplished, as far as possible, without pain and without severity.

The constitutional treatment of alopecia should consist in the adjustment and regulation of the functions of digestion and assimilation, and where no other special conditions are to be fulfilled the adoption of a tonic regimen and the administration of tonic remedies. Of these last arsenic bears the palm, and may be advantageously prescribed in doses of two to four minims three times a day, directly after food, and in any convenient vehicle.

Alopecia syphilitica will yield very readily to the treatment applicable to the parent disease, namely, iodide of potassium, with the local inunction of the nitric oxide of mercury ointment diluted in the proportion of one part to three or four of benzoated lard or vaseline, or the use of a lotion of the perchloride of mercury.

With regard to greyness, canities, or poliothrix, it is to be observed that as it depends like baldness on defective powers of the skin, the indications for treatment are exactly the same —to strengthen the part and at the same time strengthen the patient. To fulfill these indications we may resort to the same prescriptions. When cure is not anticipated, and in other cases, too, we may be asked to assist in concealing what is looked upon as a deformity.

Among temporary staining to hair he mentioned a weak solution of permanganate of potash, a lotion holding in suspension sulphur and acetate of lead, or the so-called *eau des Fées*, consisting of the hyposulphites of lead and soda; among dyes sulphides of various metals, especially silver, the pyrogallate of iron and ferro-cyanide of copper. The hair, as is well known, contains sulphur, and a solution of lead brought into contact with sulphur produces a sulphide of lead which is black in color. Sulphur and acetate of lead in suspension and solution in water supply both the elements necessary for artificial coloration of the hair, and constitute the popular lotions sold so largely.

Actual dying of the hair is a more elaborate process ; the hair must be washed with soap in the first place, to get rid of grease, which would otherwise interfere with the absorption of the fluid by the hairy tissue; secondly, the hair being dried, the metallic solution is to be employed and left to soak into the hair; and thirdly, the mordant fluid is to be brushed upon the part with a view to bring it in contact with every individual hair. If this operation sufficed for a considerable period, all would be well; but as we know that the hair grows quickly, the newly-grown part exhibits its original whiteness, and other dyeing soon becomes necessary. The tone of color produced by the first application may have been perfect, leaving nothing to be desired, but the white roots of the hair cannot be reached without a fresh coloring being diffused over the whole, and then the evils become apparent; a succession of coats of color render the hair more intensely black than nature herself could have accomplished, and the harmony of the featurs of the individual is disturbed : the mellowing of the lineaments of the countenance produced by white hair is reversed by the depth of the blackness, and the features are rendered harsh and severe. The theory that an appearance of youth is maintained by the color of the hair is not consistent with fact, and there is always the danger that the hair may appear youthful, while the features themselves are expressive of old age.

As to danger to the health and constitution from dyeing the hair, Professor Wilson thinks that we cannot reasonably allege the possibility of any serious evils, for lead, to which are imputed the most dangerous of the qualities of hair dyes, enters into the composition of several of our cooling and astrigent and sedative lotions, and even injections, and although undoubtedly some cases are on record of damage resulting from its internal and excessive use, Goulard's lotion is commonly received among us as one of the most harmless of our remedies. Perhaps a distinction may be drawn between its therapeutical and its cosmetical use, but it is difficult to distinuguish the difference. It is fair, however, to state that one of our journals reports that "there died in Iowa Dr. Witherwax, from the effects of a hair-dye containing lead. He had employed the dye to his head and whiskers for four years, and during that period experienced many reminders in the shape of lead colic. Two medical men detected lead in his liver and kidneys on analysis." No further search would appear to have been prosecuted elsewhere, namely, in the direction of his drinking water. Mr. Wilson remembers two instances in which complaint was made against a lead lotion used for the purpose of coloring the hair, although several have been mentioned to him from time to time; and both cases may possibly be considered as examples of idiosyncrasy.

